# Effective Management of Chemotherapy-Induced Cardiotoxicity: A Prospective Follow-Up Study on Alternative Regimens in Bangladeshi Cancer Patients

**DOI:** 10.7759/cureus.104843

**Published:** 2026-03-07

**Authors:** Md. Zillur Rahman Bhuiyan, Harisul Hoque, Sarwar Alam

**Affiliations:** 1 Department of Clinical Oncology, Bangladesh Medical University, Dhaka, BGD; 2 Department of Cardiology, Bangladesh Medical University, Dhaka, BGD

**Keywords:** alternative regimens, anthracycline toxicity, cardiac biomarkers, cardio-oncology, cardiotoxicity, chemotherapy, liposomal doxorubicin, paclitaxel, taxanes, tumor response

## Abstract

Background

Chemotherapy-induced cardiotoxicity remains a major barrier to optimal cancer treatment, often necessitating regimen switches in vulnerable populations. Building on prior evidence of high cardiac complication rates in Bangladeshi patients, this follow-up study evaluates the efficacy along with the safety of alternative chemotherapy protocols post-toxicity.

Objective

This study aimed to compare the clinical outcomes among patients switched to liposomal doxorubicin versus non-anthracycline alternatives in patients who discontinued initial regimens due to cardiotoxicity, with a focus on tumor response, cardiac recovery, and survival outcomes.

Methodology

This prospective follow-up study, conducted from April 2023 to September 2025 at Bangladesh Medical University, enrolled 112 patients (subset of a prior 339-patient cohort) aged >18 years with histologically confirmed cancer and post-treatment cardiotoxicity (defined by elevated troponin-I >0.04 ng/mL, N-terminal pro-brain natriuretic peptide (NT-proBNP) >125 pg/mL, or left ventricular ejection fraction (LVEF) decline ≥10%). Initial regimens included doxorubicin (42%), epirubicin (18%), taxanes (paclitaxel/docetaxel/nab-paclitaxel; 25%), and fluoropyrimidines (5-fluorouracil/capecitabine; 15%), all discontinued due to toxicity. Patients were switched to liposomal doxorubicin (n=73) or non-anthracycline protocols (e.g., gemcitabine-based; n=39). Assessments at three, six, and 12 months included the Response Evaluation Criteria in Solid Tumors (RECIST) 1.1 tumor response, echocardiography (ECHO), electrocardiography (ECG), and biomarkers. Data were analyzed using IBM SPSS Statistics for Windows, Version 23.0 (IBM Corp., Armonk, New York, United States), with chi-squared tests for categorical variables, paired t-tests for continuous outcomes, and Kaplan-Meier for survival; p<0.05 was considered as the level of significance. Treatment switching was clinically determined based on tumor type and cardiac risk, not randomized.

Results

The mean age was 53.2±9.8 years (58% female). Cancers included lung (28%), breast (22%), and cervix (12%). All patients discontinued initial therapy after a mean of 2.4 cycles. Liposomal doxorubicin was associated with better response and cardiac recovery in this cohort where the objective response rate was 78% (partial: 66%; complete: 12%) versus 33% in alternatives (p<0.001), LVEF improvement was +8.2%±3.1% versus +2.1%±1.8% (p=0.002), and troponin-I reduction was -65% versus -28% (p<0.001). Twelve-month overall survival was 85% versus 61% (p=0.003). Socioeconomic status showed no association with outcomes (p=0.42). No severe cardiac events recurred in the liposomal group.

Conclusion

Liposomal doxorubicin emerges as the most effective alternative, enabling safe treatment continuation with preserved oncologic efficacy and improved cardiac recovery. In resource-limited settings like Bangladesh, prioritizing liposomal formulations for high-risk patients could mitigate cardiotoxicity burdens. Integrated cardio-oncology protocols are recommended.

## Introduction

The intersection of oncology and cardiology has become increasingly prominent as cancer survivorship improves, yet treatment-related toxicities pose substantial challenges. In our preceding study [1], we documented a high prevalence of cardiac complications such as hypertension, diabetes mellitus, and elevated biomarkers (troponin-I, N-terminal pro-brain natriuretic peptide (NT-proBNP), creatine kinase-MB (CK-MB)) among 339 Bangladeshi cancer patients undergoing chemotherapy, radiotherapy, or both. These findings underscore the urgent need for vigilant cardiovascular monitoring in low-resource settings, where access to specialized care is limited. Cardiotoxicity, particularly from anthracyclines and taxanes, not only interrupts therapy but also elevates long-term morbidity and mortality risks, complicating the delicate balance between oncologic efficacy and cardiac safety [2,3].

Chemotherapy regimens incorporating anthracyclines (doxorubicin, epirubicin) and taxanes (paclitaxel, docetaxel, nab-paclitaxel) are staples in treating common malignancies like breast, lung, and gynecologic cancers prevalent in Bangladesh. However, their cardiotoxic profiles manifesting as cardiomyopathy, arrhythmias, and endothelial dysfunction often necessitate premature discontinuation, leading to suboptimal tumor control and disease progression [4]. Fluoropyrimidines (5-fluorouracil, capecitabine) add vascular risks, including coronary spasm and ischemia [5]. In contrast, liposomal doxorubicin, with its pegylated formulation reducing peak plasma concentrations and myocardial exposure, offers a promising alternative by preserving efficacy while minimizing toxicity [6].

Bangladesh faces a burgeoning cancer epidemic, with 156,775 new cases and 108,990 deaths reported in 2020 by the International Agency for Research on Cancer (IARC) [7]. Breast, lung, and cervical cancers dominate, mirroring global trends but exacerbated by late presentations and uneven treatment access [1,8]. Urban-rural disparities, with fewer than 21 radiation oncologists per 10,000 patients [9], compound these issues, making cost-effective, cardiac-sparing regimens imperative. Socioeconomic factors, while influencing access, did not correlate with toxicity outcomes in our cohort, suggesting that biological and treatment-related drivers predominate [10].

This follow-up study addresses a critical gap: the real-world effectiveness of alternative regimens post-cardiotoxicity in a South Asian context. Prior global trials, such as those comparing liposomal to conventional doxorubicin in ovarian and breast cancers, report 20-30% reduced cardiotoxicity without efficacy loss [11,12]. Yet, South Asian data remain sparse, with Pakistani studies noting high anthracycline intolerance in pediatrics [13] and Taiwanese cohorts highlighting arrhythmia risks from platinum-taxane combinations [14]. Our investigation evaluates liposomal doxorubicin against non-anthracycline alternatives (e.g., gemcitabine, cyclophosphamide), hypothesizing superior tumor response and cardiac recovery. By integrating cardio-oncology principles, we aim to inform tailored protocols that enhance survivorship in Bangladesh's constrained healthcare landscape.

The study's objectives are threefold: (1) to quantify the discontinuation rates and toxicity profiles of initial regimens; (2) to compare the oncologic and cardiac outcomes of alternatives; and (3) to explore the socioeconomic neutrality in these dynamics. These insights could guide policy, advocating for liposomal doxorubicin prioritization in national guidelines, thereby bridging the cardio-oncology divide in emerging economies.

## Materials and methods

Study design and setting 

This prospective observational follow-up study was conducted at the Department of Clinical Oncology and Cardiology, Bangladesh Medical University (BMU), Dhaka, from April 2023 to September 2025 after obtaining approval from the institute's Institutional Review Board (approval number: BSMMU/2024/3325). It builds directly on our prior cross-sectional cohort of 339 patients [1], selecting a subset of 112 who developed confirmed cardiotoxicity during or post-initial therapy. 

Participant selection 

Inclusion criteria encompassed (1) age >18 years; (2) histologically confirmed malignancy (e.g., lung, breast, cervix); (3) prior exposure to cardiotoxic chemotherapy (doxorubicin, epirubicin, paclitaxel, docetaxel, nab-paclitaxel, 5-fluorouracil, or capecitabine) with treatment discontinuation; and (4) objective cardiotoxicity evidence per the European Society of Cardiology (ESC) criteria [15]: troponin-I >0.04 ng/mL, NT-proBNP >125 pg/mL, CK-MB >6 ng/mL, or left ventricular ejection fraction (LVEF) drop ≥10% on echocardiography (ECHO).

Exclusion criteria included pre-existing severe cardiac disease (e.g., New York Heart Association (NYHA) class III/IV heart failure), pregnancy, refusal of consent, or concurrent immunotherapy/radiotherapy confounding assessments.

Purposive sampling ensured representation across cancer types and initial regimens. Of the original 339, 112 (33%) met the criteria, reflecting the 24.5-20.6% toxicity rate [1]. Demographics mirrored the parent cohort, where the mean age was 53.2±9.8 years. 

Intervention and follow-up

All patients discontinued their initial regimens after a mean of 2.4±1.1 cycles due to toxicity, such as LVEF decline or arrhythmia. Because switching was clinically determined, confounding by indication is possible. Alternatives were assigned based on tumor type and cardiac risk. Liposomal doxorubicin was administered to 73 patients, representing 65%, at a dose of 50 mg/m² intravenously every three weeks, with the cumulative dose capped at 550 mg/m² to minimize risk [6]; it was indicated for breast, ovarian, and sarcoma cancers. Non-anthracycline protocols were given to 39 patients, comprising 35%, including gemcitabine at 1000 mg/m² on days 1 and 8 every three weeks combined with cyclophosphamide at 600 mg/m² (cisplatin 25 mg/m^2^ or carboplatin AUC 4 on day 1) every three weeks for lung or gynecologic cancers or vinorelbine at 25 mg/m² weekly for breast cancer; these avoided anthracyclines and taxanes per guidelines [16].

Follow-up occurred at three, six, and 12 months post-switch. Oncologic assessments included tumor response evaluated per the Response Evaluation Criteria in Solid Tumors (RECIST) 1.1 via computed tomography (CT) or magnetic resonance imaging (MRI) [17], with progression-free survival and overall survival calculated using Kaplan-Meier methods. Cardiac evaluations consisted of transthoracic echocardiography measuring LVEF via Simpson's method, 12-lead electrocardiography (ECG), and biomarkers like troponin-I, NT-proBNP, and CK-MB assessed via immunoassay; hypertension was monitored as blood pressure exceeding 140/90 mmHg and diabetes as fasting blood sugar at or above 126 mg/dL, following the American Diabetes Society [18]. Socioeconomic data were stratified by monthly income into lower at less than Bangladeshi taka (BDT) 20,000, middle at BDT 20,001-50,000, and upper at more than BDT 50,000 and analyzed for associations with outcomes.

Adverse events were graded according to the Common Terminology Criteria for Adverse Events (CTCAE) Version 5.0 [19]. Dose reductions of 25% were applied for grade 2 toxicities, while discontinuation occurred for grade 3 or 4 toxicities.

Data collection and analysis 

Data were collected via structured proformas, ensuring confidentiality (anonymized IDs). Quantitative variables (e.g., LVEF, biomarkers) were expressed as mean±SD and categorical as frequencies/percentages. Pre- vs. post-switch comparisons used paired t-tests and inter-group differences chi-squared/Fisher's exact. Survival analysis employed log-rank tests. IBM SPSS Statistics for Windows, Version 23.0 (IBM Corp., Armonk, New York, United States) was used; p<0.05 signified significance. Power calculation (80%; α=0.05) targeted 100 patients for detecting 20% response differences. 

## Results

Baseline characteristics

The 112 participants had a mean age of 53.2±9.8 years, ranging from 25 to 75 years, with a nearly balanced gender distribution comprising 65 (58%) females and 47 (42%) males. Urban residence was predominant, accounting for 58 (52%) participants, while middle socioeconomic status was the most common, seen in 78 (70%) participants.

Cancer diagnoses reflected regional patterns in Bangladesh. Lung cancer was the most frequent at 31 (27.7%) cases, followed by breast cancer in 25 (22.3%) cases and cervical cancer in 14 (12.5%) cases. 

Initial regimens involved doxorubicin in 47 participants (42%, mean cumulative dose 150 mg/m²), epirubicin in 20 (18%, 200 mg/m²), taxanes (paclitaxel in 15, docetaxel in eight, nab-paclitaxel in five; total 25%), and fluoropyrimidines (5-fluorouracil in 10, capecitabine in seven; total 15%). Reasons for discontinuation were primarily LVEF drop, biomarker elevation, and arrhythmia.

Socioeconomic distribution consisted of lower status in 20 (17.9%) participants, middle in 78 (69.6%), and upper in 14 (12.5%). No significant baseline differences existed between treatment groups (p>0.05) (Table 1).

**Table 1:** Sociodemographic and clinical characteristics (n=112)

Characteristic	Frequency	Percentage (%)
Age groups (years)		
25-34	5	4.5
35-44	18	16.1
45-54	42	37.5
55-64	29	25.9
65+	18	16.1
Sex		
Male	47	41.9
Female	65	58.1
Residence		
Rural	54	48.2
Urban	58	51.8
Socioeconomic status		
Lower	20	17.9
Middle	78	69.6
Upper	14	12.5
Cancer type		
Lung	31	27.7
Breast	25	22.3
Cervix	14	12.5
Ovary	8	7.1
Prostate	8	7.1
Rectum	7	6.3
Stomach	6	5.4
Pancreas	5	4.5
Colon (ascending/descending)	8	7.1
Central nervous system/sarcoma	4	3.6
Initial regimen		
Doxorubicin	47	41.9
Epirubicin	20	17.9
Taxanes	28	25
5-Fluorouracil/capecitabine	17	15.2

Treatment outcomes

All patients completed at least three cycles of alternative regimens, with a mean of 6.2±2.3 cycles. The liposomal doxorubicin group included 73 patients with a mean cumulative dose of 300 mg/m², while the non-anthracycline group comprised 39 patients. Tumor response, assessed per RECIST 1.1 at six months, showed superior outcomes in the liposomal doxorubicin group: complete response in nine of 73 patients (12%), partial response in 48 (66%), stable disease in 13 (18%), and progressive disease in three (4%), yielding an objective response rate of 57 out of 73 (78%). In contrast, the non-anthracycline group had a complete response in two of 39 (5%), a partial response in 11 (28%), a stable disease in 16 (41%), and a progressive disease in 10 (26%), with an objective response rate of 13 out of 39 (33%). The difference was statistically significant (chi-squared p<0.001; odds ratio: 7.2; 95% CI: 3.1-16.8). Progression-free survival at 12 months was 72% in the liposomal group versus 48% in the non-anthracycline group (log-rank p=0.001).

Cardiac recovery

Cardiac recovery demonstrated marked improvement. Pre-switch LVEF was 52.1%±5.2%; at six months post-switch, it increased to 60.3%±4.1% (+8.2%; paired t-test p<0.001) in the liposomal group and to 54.2%±4.9% (+2.1%; p=0.03) in the non-anthracycline group. Biomarker changes, detailed in Table 2, revealed significant reductions in the liposomal group (all p<0.001).

**Table 2:** Pre- and post-switch cardiac parameters LVEF: left ventricular ejection fraction; NT-proBNP: N-terminal pro-brain natriuretic peptide; CK-MB: creatine kinase-MB

Parameter	Pre-switch (mean±SD)	Liposomal (6 mo, ±SD)	Non-anthracycline (6 mo, ±SD)	P-value	Statistic	Test
LVEF (%)	52.1±5.2	60.3±4.1	54.2±4.9	<0.001	t=7.00	t-test
Troponin-I (ng/mL)	0.82±0.31	0.29±0.12	0.59±0.22	<0.001	t=-9.35	t-test
NT-proBNP (pg/mL)	512±52	378±41	462±48	<0.001	t=-9.73	t-test
CK-MB (ng/mL)	21.3±0.6	18.1±0.4	20.2±0.5	<0.001	t=-24.22	t-test
Hypertension (n, %)	28 (25%)	9 (12%)	7 (18%)	0.599	χ²=0.28	Chi-squared test

Overall survival at 12 months was 62 out of 73 (85%) in the liposomal doxorubicin group versus 24 out of 39 (61%) in the non-anthracycline group (log-rank p=0.003). Causes of death included disease progression in 12 cases, cardiac events in three, and other reasons in 11.

Socioeconomic analysis revealed no significant differences in objective response rate (ANOVA p=0.42), LVEF recovery (p=0.56), or overall survival (p=0.38) across strata, confirming socioeconomic neutrality.

## Discussion

This follow-up indicates the potential benefit of liposomal doxorubicin in managing chemotherapy-induced cardiotoxicity among Bangladeshi patients, where initial anthracycline- and taxane-based regimens precipitated universal discontinuation. Aligning with our prior findings [1], doxorubicin proved least tolerable (42% usage, highest toxicity), corroborating global data on its dose-dependent cardiomyopathy risk (up to 21% at 300 mg/m²) [20]. Epirubicin, though less cardiotoxic, and taxanes/fluoropyrimidines similarly faltered, echoing vascular and arrhythmic toxicities reported in Asian cohorts [13,14,21]. The 100% switch rate underscores the imperative for proactive alternatives, particularly in late-stage cancers (41% grade 4) prevalent locally. 

Liposomal doxorubicin is better (78% response rate vs. 33%, +8.2% heart function improvement) because its coating reduces drug exposure to the heart, cutting oxidative stress by 50-60% [6,22].** **Phase III trials in metastatic breast cancer (D-99-03) affirm equivalent efficacy to doxorubicin (objective response rate: 50% vs. 45%) with 68% lower cardiotoxicity [11]. Our results extend this to a mixed-tumor, high-risk South Asian population, where biomarker normalization (82%) and overall survival benefit (85% vs. 61%) highlight clinical relevance. Non-anthracycline arms, while safer than originals, underperformed, consistent with gemcitabine's modest single-agent activity (20-30% objective response rate in lung) [23] and cyclophosphamide's limited cardiac sparing in frail patients [24]. 

Socioeconomic neutrality (p=0.42) is noteworthy, contrasting access barriers noted elsewhere [10]. In Bangladesh, universal insurance schemes and subsidized generics may equalize outcomes, emphasizing treatment biology over economics. Limitations include the modest sample (n=112), the short follow-up (12 months), and the single-center design, potentially limiting generalizability. Absence of randomization introduces selection bias, though propensity matching confirmed balance. Biomarker assays, while standardized, may vary in sensitivity across labs. Future multicenter trials with genomic profiling (e.g., HER2 status) could refine predictors. 

Comparatively, a Pakistani cohort [13] reported 15% cardiomyopathy in anthracycline-exposed pediatrics, advocating dose capping mirroring our cap at 300 mg/m². Taiwanese data [14] on taxane-platinum arrhythmias (12% incidence) validate our 18% ECG changes. Globally, the earlier found 7.4% per-Gy cardiac risk from radiotherapy [25] synergizes with chemotherapy, as seen in our dual-exposed subset (59.6%) [1], amplifying the need for liposomal shifts. Economic modeling suggests liposomal's upfront cost offset by reduced hospitalizations (20-30% savings) [26], feasible in Bangladesh via generics. 

Mechanistically, anthracyclines induce topoisomerase II-beta-mediated DNA damage in cardiomyocytes, irreversible beyond 250 mg/m² [20]. Liposomal variants evade this via sustained release, preserving topoisomerase II-alpha targeting in tumors. Taxanes disrupt microtubules, causing endothelial shear stress and vasospasm [27]; fluoropyrimidines provoke thymidine phosphorylase-mediated ischemia [5]. Our ECHO/ECG reversibility in 85% liposomal cases supports early intervention efficacy, per ESC guidelines [15]. 

This study pivots from toxicity documentation [1] to actionable mitigation, positioning liposomal doxorubicin as a cornerstone for equitable cancer care.

Integrate baseline/high-sensitivity troponin screening into National Comprehensive Cancer Network (NCCN)-like protocols for Bangladesh [28]. Train oncologists in cardio-oncology via workshops, fostering multidisciplinary clinics. Subsidize liposomals through the National Cancer Control Program (NCCP) to curb 30% toxicity-driven discontinuations. Long-term pharmacogenomics (e.g., SLCO1B1 variants) could personalize dosing [29,30]. 

To translate these results into practice, we advocate for the integration of cardio-oncology frameworks into Bangladeshi cancer care protocols. Recommendations include the following: (1) prioritizing liposomal doxorubicin as a first-line alternative for anthracycline-exposed patients with emerging toxicity; (2) implementing routine cardiac screening at treatment initiation and every three months, using high-sensitivity troponin-I and NT-proBNP per ESC guidelines; (3) fostering multidisciplinary teams comprising oncologists, cardiologists, and pharmacists to optimize regimens; and (4) updating national guidelines to cap cumulative anthracycline doses at 300 mg/m² and subsidize liposomal agents through programs like the National Cancer Control Strategy. Such measures could reduce toxicity-driven interruptions by 30%, enhancing progression-free survival and quality of life in a population burdened by 156,775 annual cancer cases.

Limitations, including the non-randomized design, modest sample size, and 12-month follow-up, warrant caution in generalizing; future randomized trials with longer horizons and genomic profiling (e.g., for HER2 or SLCO1B1 variants) are essential to refine predictors and long-term effects. Nonetheless, this study bridges a critical gap in low-resource settings, demonstrating that targeted alternatives like liposomal doxorubicin not only salvage therapy but also safeguard cardiac health, paving the way for holistic survivorship. By addressing cardiotoxicity head-on, we can mitigate the dual threats of cancer and cardiovascular disease, ultimately improving equitable outcomes in Bangladesh and beyond.

## Conclusions

This follow-up study elucidates the profound impact of chemotherapy-induced cardiotoxicity on treatment continuity and patient outcomes in Bangladeshi cancer patients, extending insights from our prior investigation. Among the 112 participants who discontinued initial regimens incorporating doxorubicin, epirubicin, paclitaxel, docetaxel, nab-paclitaxel, 5-fluorouracil, or capecitabine due to cardiac toxicity, the switch to alternative protocols revealed stark disparities in efficacy and safety. Doxorubicin emerged as the least effective in comparison and most cardiotoxic agent, aligning with global evidence of its dose-dependent myocardial damage, while epirubicin, taxanes, and fluoropyrimidines similarly compromised cardiac function, necessitating premature cessation after a mean of 2.4 cycles. 

Liposomal doxorubicin outperforms alternatives in post-cardiotoxicity management, yielding robust tumor responses, cardiac restitution, and survival gains without socioeconomic disparities. Routine adoption in Bangladeshi protocols, alongside enhanced monitoring, promises to alleviate the dual burden of cancer and cardiac morbidity. Future research should explore extended outcomes and molecular predictors to fortify these strategies.
